# Comparing factors associated with overall satisfaction for different forms of remote breastfeeding support in the UK

**DOI:** 10.1186/s13006-024-00641-0

**Published:** 2024-05-22

**Authors:** Gill Thomson, Marie-Clare Balaam, Svetlana Tishkovskaya

**Affiliations:** 1 School of Nursing and Midwifery, University of Central Lancashire, Preston, UK; 2 Health Statistics Lancashire Clinical Trials Unit, University of Central Lancashire, Preston, UK

**Keywords:** Breastfeeding, Infant feeding, Advice, Helpline, Social media, Survey, Evaluation

## Abstract

**Background:**

Remote forms of breastfeeding support, such as helplines and social media, offer a flexible and convenient form of support to offer help at critical points, e.g., when the risk of breastfeeding cessation is high. Currently, there is little known about who accesses different forms of remote breastfeeding support and what factors impact overall satisfaction. As part of an evaluation of the UK National Breastfeeding Helpline (NBH) (which offers breastfeeding support via a helpline and online media), we aimed to (a) understand who accesses different forms of NBH support, and (b) identify key factors associated with overall satisfaction for helpline and online media support.

**Methods:**

All service users who contacted the NBH between November 2021 and March 2022 were invited to participate in the evaluation via an online survey. Survey questions explored the type and timing of support, reasons for the contact, attitudes towards the help and support received, impact of the support on breastfeeding experiences and demographic factors. Chi-squared and Mann–Whitney tests explored variations in who accessed the helpline or online media. Multiple linear regression models were fitted to explore the factors related to the service users’ ‘overall satisfaction’. The quantitive data were combined with qualitative comments into descriptive themes.

**Results:**

Overall, online media users were significantly more likely to be younger, White, multiparous, less educated and have English as a first language compared to those who contact the helpline. Similar factors that significantly influenced overall satisfaction for both support models were the service being easy to access, receiving helpful information that met expectations, resolving breastfeeding issues, and feeling reassured and more confident. Significant factors for the helpline were callers feeling understood and more knowledgeable about breastfeeding following the call, being able to put into practice the information provided, feeling encouraged to continue breastfeeding, feeling that the volunteer gave the support that was needed, and seeking out additional support.

**Conclusions:**

Online and helpline forms of breastfeeding support suit different demographics and call purposes. While optimal breastfeeding support needs to be accessible, flexible and instrumental, helpline users need real-time relational support to deal with more complex challenges.

## Background

Telephone communication is a key feature of UK health services [1]. Helplines provide flexible access to expert advice and information [2] and are considered low-cost [3]. Telephone support can reduce key barriers to healthcare such as accessibility through the offer of a potentially confidential and non-stigmatizing form of support [4–6]. During the COVID-19 pandemic, social distancing measures meant that virtual support became even more relevant and essential [7]. A Cochrane review found that telephone support is beneficial for supporting breastfeeding [8]. As women can experience a lack of breastfeeding support from universal care [9], helplines are considered to offer a flexible and convenient form of supplementary support.

While breastfeeding helplines offer a reactive form of support, they can enhance mothers’ confidence and provide immediate help, including at times when other services are unavailable [4]. This means that women can access support at critical points when the risk of breastfeeding cessation is high [10]. Gallegos and colleagues studied the calls made to a 24-hour parenting helpline in Australia to identify characteristics of calls that support breastfeeding self-efficacy [10]. This study found that interactional characteristics that promoted self-efficacy were ‘privileging the mother’, teamwork and credible affirmation. Factors that appeared to undermine self-efficacy were laissez-faire affirmation and pragmatic problem-solving [10]. An evaluation of a UK-based breastfeeding helpline service found that 74.6% of callers were very satisfied, and 19.8% were satisfied with the help and support received [4]. Multiple regressions of the evaluation data revealed key factors associated with overall satisfaction related to: volunteers having sufficient time to deal with the callers’ issues, the information being perceived as helpful, the volunteers providing the support the callers needed, and callers feeling reassured following the call [11]. While an analysis of qualitative feedback from the same evaluation data [4] mirrored these positive findings, some critical issues, albeit from a small number of callers, were also highlighted. These relate to complaints by some callers about delays in their calls being answered, and dissatisfaction with volunteers’ inability to answer their questions or offer new insights into their breastfeeding issues, which may reflect peer training, confidence and capacity to provide support remotely [4].

In more recent years, breastfeeding support has been provided via web-based technologies. For example, via access to a closed online forum [12, 13], or a website generating personalised smartphone notifications [14]. There have been some evaluations of closed online/Facebook groups [15, 16] and text support [17]: these studies identify similar positive features to helpline support, with women valuing the authentic presence of trained and lay peers [15]. A relatively new feature used in breastfeeding and healthcare more generally is web chat. Web chat involves real-time opportunities to seek information and help via an online platform.

Since February 2008 in the UK, a National Breastfeeding Helpline (NBH) has been in operation provided by two national breastfeeding organisations – the Breastfeeding Network (BfN) and the Association of Breastfeeding Mothers (ABM). The NBH offers support provided by volunteer peer supporters, via different modalities. First, there is the helpline that offers support through a national number, with calls charged at local rates. The helpline service is available in England, Wales, Scotland and Northern Ireland, with lines open from 9.30am until 9.30pm every day. From March 2020 there has been an option to leave a voicemail if there is no one available to answer their call, with the intention to provide a call-back within 24 h. Helpline support is also available in Welsh, Polish, Bengali and Sylheti. The service also provides support (during the same daytime hours as the helpline) via web chat and online media. Web chat is a synchronous form of support offered at set times (that varied, depending on volunteer availability) involving volunteers responding to callers’ queries via a web chat function on the NBH website. Online media is asynchronous with volunteers answering information requests posted on the NBH’s or BfN’s Facebook or Instagram accounts as and when they are available. Current data reports that each month there are between 600 and 1,000 calls answered by the helpline (with the number of calls received being approximately the same), 170–250 voice mails returned, 450–550 online media requests, and 20–60 web chats. While the NBH has over 300 registered volunteers, there are generally ~ 100 + active each month, with an equal split between volunteers from the ABM and the BfN. The volunteer peer supporters are individuals who have had their own experiences of breastfeeding and have received accredited training via their respective organisations. Volunteers can choose which forms of support they want to provide (helpline, web chat, online media), and while asked to answer 100 helpline calls each year, this is not mandated. The volunteers receive no direct reimbursement, but childcare can be paid as needed. Providing support via the helpline incurs no direct costs for volunteers, and if needed a low-cost pay-as-you-go mobile is provided.

An associated NBH service (provided by the BfN) is the Drugs in Breastmilk (DIBM) information service which was established in 2007 due to a high frequency of NBH calls relating to medication use and breastfeeding. While DIBM used to operate as a helpline, support is now offered via email or Facebook messenger. The service is provided by a team of pharmacists, with between 250 and 400 enquiries each month. Women who call the NBH with a medication-related concern are directed to this service. All NBH services (which includes the DIBM) provide information, instrumental and emotional support and where appropriate, signpost service users through to other sources of help and support.

An evaluation of the NBH service was undertaken in 2011 [4, 11]. As it has been almost a decade since the previous evaluation, and the NBH, in line with how consumers access health-related information and support, has moved towards other online media options (social media, web chat), a further evaluation was commissioned by the NBH. In this paper, we report on two of the key aims of the evaluation in terms of identifying variations in terms of who accesses the different forms of NBH support, and to compare factors associated with overall satisfaction between those who called the helpline and those who used online media.

## Methods

### Aim

In this paper, we compare different forms of NBH support - helpline and online media (i.e., social media and DIBM). As web chat provides a different form of support to the other online options, and is not widely used, we focus on social media and DIBM only. Here we address two key aims to: (a) understand who accesses different forms of NBH support, and (b) identify key factors associated with overall satisfaction for helpline and online media breastfeeding support. To our knowledge, this is the first study to compare and explain different types of breastfeeding support within one service.

### Design

Similar to the previous evaluation, an exploratory cross-sectional survey study was undertaken utilising quantitative and qualitative data [11].

### Data collection

An online confidential survey (hosted by Qualtrics) was developed using questions from the 2011 evaluation [4, 11], and additional questions to capture how service users found out about the different types of support, and why individuals opted for different forms of support. The survey included questions regarding the type and timing of support, reasons for the contact, attitudes towards the help and support received, impact of the support on breastfeeding experiences, other personal benefits, follow-up actions, final reflections and demographic/personal details. Open text sections and Likert scales were used, e.g. scales of 1 (extremely dissatisfied, strongly disagree) – 5 (extremely satisfied, strongly agree), and often included a ‘not applicable’ option as appropriate. The survey was reviewed by five members of the NBH for acceptability and comprehensiveness.

### Recruitment

The survey was distributed over four months (between 15th November 2021 to 15th March 2022). To encourage inclusivity and in line with the language lines provided by the NBH, two translated versions (one in Bengali and Polish) were issued over a restricted period (15th March – 15th April 2022). This restricted time was related to delays in translations being organised and completed within the NBH service.

Depending on what type of support the service user had accessed (helpline or virtual), the survey was distributed in different ways.

**Fig. 1:**
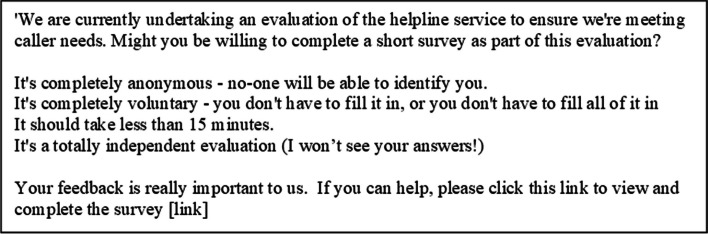
Information forwarded to online media users

- Helpline – at the end of the contact, callers were asked if they would be willing to receive information about the evaluation - if they agreed, their names and emails were recorded on a Microsoft Form (shared with the evaluation team). The research staff then sent the caller a link to the survey. The existing NBH call record was adapted to record this detail. The call record is expected to be completed after every contact to capture basic details including who called (i.e., breastfeeding parent, partner, not disclosed), age of child, reason for the call, how distressed the caller sounded, and first half of the caller’s postcode. Caller information –whether the caller had called the helpline before and how they heard about the helpline and demographic information (ethnicity, age they completed full-time education, whether English is their first language, age) – is meant to be routinely collected from one in five callers. Over the evaluation period, volunteers were requested to collect this information from all callers to help elicit whether we captured a representative sample.
- Online support - To prevent unnecessary transfer of confidential information, and as online media users tended to leave the contact as soon as the question(s) had been answered, it was intended that *every* online media user who contacted these services over the evaluation period would be routinely sent details of the evaluation and a link to the survey (see Fig. 1). The NBH do not routinely collect caller-related/demographic information on online media users, so additional information was not collected.

Pilot: Over the first 2 weeks of data collection, the online survey was piloted to assess data collection procedures (102 survey responses received). Following this period, it was noted that several participants were completing all/almost all the questions on the online platform but were not clicking ‘submit’ (originally an ethics requirement). To prevent data wastage, the documentation was revised to inform participants that all data would be used even if the ‘submit’ button was not clicked. This change was agreed with the funders and the ethics committee.

### Data analysis

Descriptive statistics (frequencies and percentages for categorical data - including Likert-type responses) were undertaken for all variables. Inferential methods (regression, association and two group comparison tests and 95% confidence intervals) were also undertaken using the statistical packages SPSS v.28 and Stata 17.0. The significance level was set to 5%.

Chi-squared tests for categorical variables and Wilcoxon rank-sum (Mann–Whitney) test for variables measured on Likert scale were undertaken to compare the service users’ responses across the two support groups (helpline and online media): with comparisons made between service user characteristics and call/contact characteristics to help understand variations in who accessed the different forms of support and how easy (or difficult) it was to access the different support modalities.

Multiple linear regression models were fitted to explore potential explanatory factors related to the service users’ ‘overall satisfaction’ towards the support received via the NBH. Overall satisfaction was measured using the question ‘How would you rate your overall satisfaction with the NBH support?’ scored on a scale of 1- Extremely dissatisfied – 5-Extremely satisfied. Modelling was conducted using hierarchical multiple regression analysis that allows specifying a fixed order of entry for variables to control for the effects of covariates. Specifically, a two-level linear modelling was applied in a hierarchical manner in which service user characteristics and call/contact characteristics were considered at the lowest level of the hierarchy constituting Model 1 (and with initial significant factors included in the subsequent models). Thereafter, other factors were considered at the second level of the hierarchy, with service user views on service characteristics (Model 2), influence of support on individual service users’ breastfeeding experiences (Model 3), and service user wellbeing and follow-up support variables (Model 4) being considered in separate models. At each level, model selection was initially via a backward elimination process which included factors at that level, with all significant terms from the lower level of the hierarchy being included in all three models considered at higher levels of the hierarchy. A 5% significance level was used for inclusion and exclusion of factors in the backward elimination. For the model chosen (at each level of the hierarchy), 95% confidence intervals and adjusted mean difference for satisfaction score were presented for the effects of each of the factors remaining in the model, that is for statistically significant variables. The modelling was done separately for service users who received the support via the helpline or online media (where social media and DIBM were combined). This was to determine whether there were any key differences in how support was perceived and experienced across the two main support modalities (helpline or online media). The web chat data were excluded from the analysis as the service was limited and not representative of online media support.

All qualitative data from the open-ended questions were entered into a qualitative software package (MAXQDA). A basic content analysis was used to organise the data into descriptive codes and basic themes, similar to other mixed-method studies [18], whereby we used the qualitative data to substantiate and help explain the quantitative findings (e.g., variations in demographics and factors underpinning overall satisfaction) as appropriate. The first author (GT) developed the themes, which were then refined through discussion with MCB; the final themes were reviewed and validated by the third author (ST).

## Results

While some 1,142 started the survey, a total of 1,126 participants completed at least one of the survey questions: 1,078 questionnaires were from service users who had contacted the helpline or online media (social media or DIBM), with 48 web chat responses excluded. Five of the translated versions were started (two Polish and three Bengali), but as none of the survey questions were answered, they were removed from the final data set. The number of calls/contacts to the NBH services over the evaluation period and how many surveys were completed for each different type of support are presented in Table 1.

**Table 1:** Response rates by types of support

NBH service^a^	Number of calls/contacts	Completed questionnaires
Helpline	3,566^b^	552
Social media	2,189	478
Drugs in Breastmilk	1,374	48
**Total**	**7,129**	**1,078**

^a^While web chat has not been included, over the evaluation period there were 107 web chat conversations, with 48 questionnaires completed by those who used the web chat service

^b^844 of these were voicemail (2,722 calls direct to the helpline)

While overall the data suggests that the views of ~ 15% of service users had been captured (1,078/7,129), the NBH figures represent *calls/contacts* and not the number of service users. While ongoing online media conversations or helpline calls (e.g., if call is interrupted) are recorded as one contact, it was not possible to formally record a denominator of individual service users, as e.g., they may have re-contacted the NBH services about a different issue over the evaluation period. From our sample, 724 (67.16%) stated that this was their first contact (five could not remember (0.46%), two did not answer this question (0.19%)), and ~ 32% of participants had previously contacted NBH services.

Further analysis was undertaken to compare the demographics (age, ethnicity, education level) of those who took part in the evaluation (helpline only), with those who contacted the helpline service over the evaluation period. While it was intended that all callers to the helpline would be asked for demographic-related information (rather than the usual one in five callers), there was ~ 35% of data missing (related to callers wanting to remain anonymous, and/or how the contact was ended). Overall, however, there were similar percentages of callers who were White or non-White (White 84.2% vs. 77.1%), who were educated to GCSE or below or A level of above (GCSE or below 3.6% vs. 4.0%), and who were aged either below or above 35 years (below 35 years 61% vs. 68%) between the two groups (the percentages are the evaluation participants vs. general helpline service users where missing data were excluded), thereby indicating that the evaluation sample was generally representative of the wider population.

Overall, 942 (97.5%) (missing data removed) were extremely satisfied/satisfied with the support received, with similar levels of satisfaction reported across the three different types of support (helpline, social media, DIBM). Most service users contacted the NBH services about a ‘specific difficulty’ (*n* = 676; 62.71%), just under 9% made contact for ‘general support’ (*n* = 93; 8.63%), and 239 (22.17%) for both (70 (6.49%) of participants did not answer this question). When considering this question separately for those who accessed the helpline or online media, a slightly higher percentage contacted online media for a specific difficulty (73.84% (*n* = 367) vs. 60.47% (*n* = 309)). Most service users involved in the evaluation had contacted the service about an issue they were personally facing (*n* = 1042; 96.66%), with the remaining (*n* = 27, 2.50%) calling on behalf of their partner (*n* = 8), friend/family member (*n* = 5), client (*n* = 6), or other (*n* = 8) such as healthcare professionals.

Below we present the descriptive and inferential statistics. An interpretation of these findings contextualised by qualitative comments is then presented in key descriptive themes.

### Comparing service user and call/contact characteristics

The descriptive statistics for service user characteristics and call/contact characteristics are presented in Table 2, and the Chi-squared tests, and Wilcoxon rank-sum (Mann–Whitney) tests that compare these variables across the two support options are detailed in Table 3.

**Table 2:** Descriptive statistics for servicer user characteristics and call/contact characteristics (frequency (%) unless otherwise stated)

Characteristics	Helpline *n* = 552 (51.2%)	Online Media *n* = 526 (48.8%)	Total *n* = 1078
**Service user characteristics**	Frequency (%)		
Age			
under 24	8 (1.5%)	22 (4.2%)	30 (2.8)
25–34	290 (52.5%)	292 (55.5%)	582 (54.0%)
over 35	190 (34.4%)	148 (28.1%)	338 (31.4%)
missing	64 (11.6%)	64 (12.2%)	128 (11.9%)
Number of children, median (range)	2 (2–5)	2 (1–8)	2 (1, 8)
First time mother			
Yes	340 (61.6%)	259 (49.2%)	599 (55.6%)
No	141 (25.5%)	194 (36.9%)	335 (31.1%)
missing	71 (12.9%)	73 (13.9%)	144 (13.4%)
Age of child at time of call/contact			
0–2 weeks	101 (18.3%)	22 (4.2%)	123 (11.4%)
over 2 weeks − 8 weeks	159 (28.8%)	93 (17.7%)	252 (23.9%)
over 8 weeks − 6 months	101 (18.3%)	114 (21.7%)	215 (19.9%)
over 6 months − 1 year	66 (12.0%)	89 (16.9%)	155 (14.4%)
over 1 year − 2 years	40 (7.3%)	86 (16.4%)	126 (11.7%)
over 2 years	10 (1.8%)	42 (8.0%)	52 (4.8%)
not recorded^a^	11 (2.0%)	16 (3.0%)	27 (2.5%)
missing	64 (11.6%)	64 (12.2%)	128 (11.9%)
Feeding method			
exclusive breastfeeding/breastmilk	256 (46.4%)	205 (39.0%)	461 (42.8%)
mixed feeding (breast and formula)	119 (21.6%)	61 (11.6%)	180 (16.7%)
formula feeding	8 (1.5%)	3 (0.6%)	11 (1.0%)
complementary foods (child receiving breastmilk and other foods)	88 (15.9%)	184 (35.0%)	272 (25.2%)
exclusively solids (child eating solid foods, no breastmilk provided)	14 (2.5%)	2 (0.4%)	16 (1.5%)
don’t know	2 (0.4%)	5 (1.0%)	7 (0.7%)
missing	65 (11.8%)	66 (12.6%)	131 (12.2%)
Breastfed older children			
yes	107 (19.4%)	147 (28.0%)	254 (23.6%)
no	42 (7.6%)	55 (10.5%)	97 (9.0%)
not applicable	328 (59.4%)	253 (48.1%)	581 (53.9%)
don’t know	1 (0.2%)	5 (1.0%)	6 (0.6%)
missing	74 (13.4%)	66 (12.6%)	140 (13.0%)
Education			
GCSE (or equivalent)	17 (3.1%)	41 (7.8%)	58 (5.4%)
A level (or equivalent)	57 (10.3%)	85 (16.2%)	142 (13.2%)
degree	214 (38.8%)	173 (32.9%)	387 (35.9%)
postgraduate degree	179 (32.4%)	127 (24.1%)	306 (28.4%)
other^b^	15 (2.7%)	24 (4.6%)	39 (3.6%)
don’t know	2 (0.4%)	6 (1.1%)	8 (0.7%)
missing	68 (12.3%)	70 (13.3%)	138 (12.8%)
Marital status			
married	319 (57.8%)	276 (52.5%)	595 (55.2%)
living with partner	147 (26.6%)	144 (27.3%)	291 (27.0%)
in relationship	3 (0.5%)	12 (2.3%)	15 (1.4%)
single	13 (2.4%)	21 (4.0%)	34 (3.2%)
other^c^	4 (0.7%)	6 (1.1%)	10 (0.9%)
missing	66 (12.0%)	67 (12.7%)	133 (12.3%)
Ethnicity			
White	409 (74.1%)	424 (80.6%)	833 (77.3%)
Asian	41 (7.4%)	17 (3.2%)	58 (5.4%)
Black	7 (1.3%)	1 (0.2%)	8 (0.7%)
Mixed	19 (3.4%)	14 (2.7%)	33 (3.1%)
Other	10 (1.8%)	3 (0.6%)	13 (1.2%)
missing	66 (12.0%)	67 (12.7%)	133 (12.3%)
English as a first language			
yes	432 (78.2%)	430 (81.8%)	862 (80.0%)
no	56 (10.1%)	30 (5.7%)	86 (8.0%)
missing	64 (11.6%)	66 (12.6%)	130 (12.1%)
**Call/contact characteristics**			
Number of times callers had previously contacted the NBH services, median (IQR)	2 (1, 3)	2.5 (1, 5)	2 (1, 4)
How easy did you find it to get through to a volunteer / to use the NBH social media service?			
difficult^d^	58 (10.5%)	4 (0.8%)	62 (5.8%)
neither easy nor difficult	56 (10.1%)	9 (1.7%)	65 (6.0%)
somewhat easy	111 (20.1%)	35 (6.7%)	146 (13.5%)
extremely easy	299 (54.2%)	453 (86.1%)	752 (69.8%)
missing	28 (5.1%)	25 (4.8%)	53 (4.9%)

^a^ “not recorded” includes cases where the caller is pregnant, no baby, tandem feeding

^b^ Callers cited a wide range of qualifications such as NVQs, Higher National Diplomas, postgraduate diplomas, and doctorates

^c^ Includes civil partnership, separated, or unsure

^d^ ‘Extremely difficult’ and ‘Somewhat difficult’ were combined in the category ‘Difficult’ due to the category ‘Extremely difficult’ being very lowly populated across both types of support

**Table 3:** Comparing service user characteristics and call/contact characteristics between helpline callers and online media users

Variable	Helpline *n* (%)	Online Media *n* (%)	*p*-value
**Service user characteristics**^a^			
Is English your/their first language? (Yes)	432 (88.5%)	430 (93.5%)	0.008
First-time mother? (Yes)	340 (70.7%)	259 (57.2%)	< 0.005
White ethnicity (vs. non-white)	409 (84.2%)	424 (92.4%)	< 0.005
Level of education			< 0.005
GCSE (or equivalent)	17 (3.6%)	41 (9.6%)	
A Level (or equivalent)	57 (12.2%)	85 (20.0%)	
degree	214 (45.8%)	173 (40.6%)	
postgraduate degree	179 (38.3%)	127 (29.8%)	
Age			0.004
under 24	8 (1.6%)	22 (4.8%)	
25–34	290 (59.4%)	292 (63.2%)	
over 35	190 (38.9%)	148 (32.0%)	
**Call/contact characteristics**			
Was this the first time you had used these services? (Yes)^a^	410 (75.1%)	314 (59.8%)	< 0.005
How easy it was to get through to a volunteer^b^, ^c^	4.2 (1.0)	4.9 (0.4)	< 0.005

^a^Chi-squared test

^b^Wilcoxon rank-sum (Mann–Whitney) test

^c^Agreement was measured on Likert scale with 1 for Extremely difficult, 2 Difficult, 3 Neither easy nor difficult, 4 Somewhat easy, 5 Extremely easy

### Factors that influence overall satisfaction

Similar to the 2011 evaluation [11] and to make the results comparable, the impact of service user characteristics and call/contact characteristics on overall satisfaction were investigated first (Model 1), as these were not influenced by the experiences during the call. Service user characteristics included were age (re-categorised into three categories “under 24”, “25–34” and “over 35”), ethnicity (re-coded as “White” and “non-White”), whether the service user was a first-time mother, whether mother had breastfed before, whether English was a first language, and level of education (classified as GSCE or equivalent, A level or equivalent, degree, postgraduate degree). Call/contact characteristics included whether it was the first time they had used the NBH services and service user attitudes towards (a) how easy/difficult it was for their call to be answered (for helpline) or (b) how easy/difficult it was to use the NBH support (for online media). The participants’ responses of “extremely difficult” and “somewhat difficult” were combined to “difficult” due to “extremely difficult” being very lowly populated across all types of support.

After identifying significant factors in Model 1, the regression model was expanded to explore how service user views’ on service characteristics (attitudinal responses to the help and support they received) affected overall satisfaction (Model 2), to explore the influence of support on individual service users’ breastfeeding experiences on overall satisfaction (Model 3), and whether overall satisfaction was influenced by the service users’ wellbeing (feeling less worried, less stressed, more confident, reassured and more knowledgeable about breastfeeding) and whether follow-up support options were provided (Model 4).

Modelling results for all four models for the helpline and online media support are summarised in Table 4. The table also reports adjusted R-square statistics as an unbiased estimator of R-square in the population [19]. The adjusted R-square was consistently higher for the helpline than for online media across all the models which means that the variables in the helpline model explain more of the variability in overall satisfaction than the online media data. This finding may relate to telephone conversations being more focused and detailed than virtual contact. Alternatively, it may demonstrate that the online audience is more heterogeneous compared to helpline users. Also, Table 4 shows that Model 2 has the highest adjusted R-square compared to other models: this demonstrates that the service characteristics variables explain more of the variability in satisfaction than factors in other models.

**Table 4:** Results of regression modelling factors explaining overall satisfaction for helpline and online media responders

Characteristic	Helpline^a^		Online Media^a^	
	Adjusted mean difference for satisfaction score (95% CI)	p**	Adjusted mean difference for satisfaction score (95% CI)	p**
**Model 1: Service user characteristics and call/contact characteristics**	**AR**^2^** = 3.1%**		**AR**^2^** = 2.0%**	
How easy did you find it to get through to a volunteer / to use the NBH online media service?				
Extremely easy				
Difficult - Extremely easy	-0.24 (-0.42, -0.06)	0.009	0.13 (-0.50, 0.76)	0.69
Neither easy nor difficult - Extremely easy	-0.04 (-0.22, 0.13)	0.64	-0.44(-0.86, 0.02)	0.04
Somewhat easy - Extremely easy	-0.13 (-0.27, 0.00)	0.05	-0.30(-0.51, -0.08)	0.006
English first language, Yes - No	0.28 (0 0.12, 0.45)	0.001		
**Model 2: Service characteristics**	**AR** ^**2**^ **= 32.4%**		**AR** ^**2**^ **= 26.6%**	
How easy did you find it to get through to a volunteer / to use the NBH online media service?				
Extremely easy				
Difficult - Extremely easy		0.43		0.95
Neither easy nor difficult - Extremely easy		0.97		0.26
Somewhat easy - Extremely easy		0.63		0.75
English first language, Yes - No	0.27 (0.13, 0.41)	< 0.001		
I liked being able to receive support from another breastfeeding parent.		0.21		0.065
I liked receiving support from someone who doesn’t know me.		0.64		0.67
I felt listened to and was given the opportunity to explore my concerns		0.08		0.14
I found it easy to share breastfeeding issues during the contact.		0.34		0.93
I felt that the volunteer had enough time for me.		0.81		0.75
The information the volunteer provided was helpful.	0.16 (0.02, 0.31)	0.03	0.29 (0.14, 0.44)	< 0.001
I felt comfortable discussing breastfeeding issues with the volunteer.		0.54		0.90
I felt that the volunteer was able to answer my questions.		0.91		0.47
The support the volunteer provided was helpful.		0.83		0.64
I felt the volunteer understood what I was talking about.	0.23 (0.09, 0.37)	0.001		0.82
I felt that the volunteer gave the support that was needed.	0.17 (0.03, 0.31)	0.017		0.18
I felt the volunteer understood how I was feeling.		0.48		0.69
I felt that the volunteer treated me with respect.		0.52		0.053
I felt that the volunteer was knowledgeable about breastfeeding issues.		0.29		0.57
I felt the volunteer respected and supported my infant feeding decisions.		0.06		0.15
The information given was personal to my situation.		0.90		0.19
The support I received met my expectations.	0.22 (0.11, 0.33)	< 0.001	0.28 (0.16, 0.40)	< 0.001
**Model 3: Influence on breastfeeding experiences**	**AR** ^**2**^ **= 13.2%**		**AR** ^**2**^ **= 6.2%**	
How easy did you find it to get through to a volunteer / to use the NBH online media service?				
Extremely easy				
Difficult - Extremely easy		0.07		0.78
Neither easy nor difficult - Extremely easy		0.93	-0.42 (-0.83, -0.007)	0.046
Somewhat easy - Extremely easy		0.23	-0.25 (-0.47, -0.034)	0.024
English first language, Yes - No	0.25 (0.09, 0.41)	0.003		
I was able to put into the practice the information provided by the volunteer	0.11 (0.02, 0.19)	0.016		0.08
The support received helped me resolve my breastfeeding issues	0.13 (0.07, 0.19)	< 0.001	0.12 (0.06, 0.17)	< 0.001
The support received encouraged me to continue breastfeeding	0.08 (0.009, 0.15)	0.028		0.29
I would not have been able to carry on breastfeeding if the NBH support service had not been contacted		0.50		0.44
**Model 4: Service user wellbeing and follow-up support**	**AR** ^**2**^ **= 28.8%**		**AR** ^**2**^ **= 18.0%**	
How easy did you find it to get through to a volunteer / to use the NBH online media service?				
Extremely easy				
Difficult - Extremely easy		0.91		0.89
Neither easy nor difficult - Extremely easy		0.91		0.10
Somewhat easy - Extremely easy		0.91	-0.21 (-0.42, -0.009)	0.041
English first language, Yes - No	0.37 (0.19, 0.55)	< 0.001		
Following contact with the NBH I felt: Less worried		0.12		0.51
Following contact with the NBH I felt: Less stressed		0.36		0.34
Following contact with the NBH I felt: More confident	0.13 (0.03, 0.23)	0.011	0.20 (0.13, 0.28)	< 0.001
Following contact with the NBH I felt: Reassured	0.25 (0.13, 0.37)	< 0.001	0.11 (0.008, 0.21)	0.034
Following contact with the NBH I felt: More knowledgeable about breastfeeding	0.14 (0.07, 0.21)	< 0.001		0.18
Following contact with the NBH I felt: More determined to continue breastfeeding		0.57		0.26
Did the volunteer encourage you to seek out additional help or support?		0.09		0.90
Did the volunteer suggest to contact the NBH service again? Yes - No	0.20 (0.08, 0.32)	0.002		0.17

^a^ Adjusted mean difference and confidence intervals have not been reported for non-significant results

** *p*-values shown (a) for non-significant factors are *p*-values when term was eliminated from the model in backward selection; (b) for significant factors are taken from final step model with only significant factors included

In the following sections, we synthesise the findings from the descriptive and inferential statistics and draw on insights from the qualitative findings to help explain the variations in findings across the two support models.

### Demographic-related issues

Overall, there were significant differences in demographic profiles between the two groups. First in terms of age (*p* = 0.004), the proportion of younger people, under 24, in the online media group was higher, while the proportion of older mothers, 35 and over, was higher in the helpline group (Table 3). The proportion of service users who were first-time mothers and educated to degree level and above was significantly higher in the helpline when compared to the online media group (*p* < 0.005). The variable ‘Is English your first language’ was associated with overall satisfaction for the helpline (*p* = 0.001) rather than online media model within the regression models (Table 4). However, when comparing this variable across the two groups, the service users who had English as a first language (*p* = 0.008) and were White British (*p* < 0.005) were more likely to use online media, and non-White, non-native English speakers were more likely to use the helpline (Table 3). These insights thereby indicate that confidence to converse in English is perhaps more essential for helpline users when compared to online media.

### Accessing the service

How ‘easy’ it was to access the service was significantly associated with overall satisfaction for the helpline and online media groups (see Table 4). Some service users referred to how the speed of response had been unexpected, ‘a*bsolutely incredible! Could not believe how quickly I received support’* particularly when contacting the NBH services out of hours or during national holidays when other support is not available:

[there was] a quick response even during the holiday period (bank holiday – Boxing Day). [Online Media/DIBM] (Participant 751).

Others complained about the need to make multiple calls to the helpline, or that waiting for responses to voicemails and online media was *‘stressful’*, with 169 (32.9%) (missing data removed) of helpline callers agreeing that the helpline opening hours should be extended. On occasion, those who called the helpline were critical about voicemails not being returned, or the support being too late to be of help:

We were not called back until the next day after which time we had more or less solved the original issue with the help of a health visitor and GP. [Helpline] (Participant 207).

When comparing the responses across the two groups, it appears that overall, significantly more online media users found accessing support easier when compared to those who called the helpline (*p* < 0.005) (Table 3). From the qualitative feedback, it is suggested that this may be due to the nature of the call/contact. Some online media users spoke of expecting a delay; with one referring to how they would have contacted ‘*a different service‘* had the issue been urgent. As those contacting the helpline are potentially more likely to request immediate support, this is likely to have tempered their views on access when timely support was not provided:

I needed the help there and then. It felt like too long to wait when you are struggling to feed your baby. [Helpline] (Participant 227).

Online media users also referred to how these sources of support meant they did not have to ‘*hold in a queue and often miss the opening hours due to working shifts‘*, thereby enabling them to seek support around their work or home life patterns:

I’ve got a 16-month old to run after who is obviously a handful! I love that the social media messages can be done around work and I’m not pressed for an instant reply. [Online Media/DIBM] (Participant 679).

Overall, first-time users were significantly more likely to use the helpline when compared to online media (*p* < 0.005). This could reflect first-time users having more ‘urgent’ issues, whereas those returning to the service may require more general help that could be resolved using asynchonous support. It may also, in part, be associated with service users’ lack of awareness of the different service offers. For example, qualitative feedback highlighted that while some service users knew about the helpline, they were unaware of the online options offered:

I only knew there was a number to call, if knew could message would of contacted NBH earlier as was nervous phoning so put it off for days. [Online Media/DIBM] (Participant 947).

Other service users reflected on how they had not been aware of the DIBM and commented that *‘It’s fantastic, although I didn’t know about it for ages!’* and ‘*I found out about the drug help on my own – wish my midwives had told me about it’.*

### Perceptions of support

Overall, there were similarities and differences in factors that influenced satisfaction between the helpline and online media models. Significant factors unique to helpline callers related to the volunteer understanding what the caller was talking about (*p* = 0.001), and the volunteer giving the support that was needed (*p* = 0.017) (see Table 4). These factors indicate a potential cause and effect of helpline callers’ feeling understood and the volunteer being able to offer needs-led care, as reflected in comments such as:

I found the person I spoke with on the hotline to be extremely helpful. It felt like I was talking to someone who just got it – who understood exactly what I was going through and gave me the most informed, supportive advice. [Helpline] (Participant 279).

Two variables that influenced overall satisfaction in both support models were the information being helpful (*p* = 0.03 helpline and *p* < 0.001 online media) and the support meeting their expectations (*p* < 0.001) (Table 4). These factors both point to how the utility of the support is crucial irrespective of how it is provided, with frequent comments praising the volunteers as ‘*professional’, ‘very well trained’*, ‘*knowledgeable’* individuals who had a ‘*breadth of knowledge’* and ‘*knew exactly what they were talking about’.* One online media user reported:

I love this service and find the information and support given surpasses that of other services. I used to spend considerable amounts of time in forums and search engines finding answers but they varied so much and we’re never straightforward. Here I get straightforward, honest responses which I know I can trust. [Online Media/Social Media] (Participant 720).

While service users referred to the helpline being the public face of the NBH service, some online media users felt that the contact still made them feel they had a personal connection with a volunteer. One commented how ‘*I felt like I was talking to a person not just a chatbot’* and another stated:

I was very surprised how quickly I received a response, and it was a proper chat. Not just one response and then done, they asked me a few questions and we had a back-and-forth conversation. [Online Media/DIBM] (Participant 801).

### Impact of support on breastfeeding

Regarding the influence of the support on service users’ breastfeeding experiences, for those who used online media, only the support helping them to resolve their breastfeeding issues was found to be a significant factor in overall satisfaction (*p* < 0.001). Several online media users referred to being given practical information such as being encouraged to ‘cluster feed’ or guidance on medication that enabled them to confidently continue to feed, which for one meant:

[I didn’t have to] choose between prioritising my own health and wellbeing through use of medications or stopping breastfeeding’ as ‘the advice meant I felt safe to continue with both’. [Online Media/DIBM] (Participant 1080).

Whereas overall satisfaction amongst helpline callers related to being able to put into practice the information provided (*p* = 0.016), and the support helping them to resolve their breastfeeding issues (*p* < 0.001) and/or encourage them to continue breastfeeding (*p* = 0.028) (see Table 4). These insights reflect that the support being able to resolve their breastfeeding challenges is crucial, and that being able to discuss the issues in real-time can be more encouraging as well as maximising the potential to translate the information into practice. One helpline caller explained the importance of:

Someone who offered real advice, and time to talk it through and help me. Issues can be difficult to resolve, and you often feel a bit isolated if it isn’t working out and having problems. They were able to really have a very open conversation and help you reduce worry and tackle the issue with their advice. [Helpline] (Participant 441).

While overall only 273 (24.24%) considered that they would not have been able to continue breastfeeding without the help of the NBH service (although not significantly associated with overall satisfaction for either group), some explicitly spoke of how contact with the NBH was crucial in providing the instrumental and/or emotional support to resolve their breastfeeding issues, and to continue breastfeeding:

The first phone call I made 10mths ago I was so done with feeding my newborn but the support the lady gave me was out of this world nearly three hours on the phone and that gave me the power to carry on. [Helpline] (Participant 231).

### Impact of support on wellbeing

Emotional and cognitive-related factors significantly associated with overall satisfaction for both helpline callers and online media users related to feeling more confident (*p* = 0.011 helpline and *p* < 0.001 online media) and reassured (*p* < 0.001 and *p* = 0.034 respectively), whereas, feeling more knowledgeable about breastfeeding was a strong significant indicator for helpline callers only (*p* < 0.001 Table 4): a difference potentially related to online media users being more likely to contact the service about a specific issue, rather than for general support and advice. Service users frequently described how important and *‘invaluable’* the support was for their mental and emotional well-being at what can be a challenging time. One helpline caller described:

I felt incredibly supported and was given words to express some of how I’d been feeling. Although I didn’t come away with much practical advice, the emotional support and time I’d been given had been invaluable. [Helpline] (Participant 320).

Increased confidence could be related to the volunteer providing information about a particular situation that ‘*put my mind at rest and gave me confidence’* to continue feeding. Or how increased knowledge enabled them to feel more empowered to talk to and challenge the advice provided by health professionals; ‘[I felt] *more informed when talking to GP, armed with information’*. Following contact with the NBH, service users spoke of feeling more ‘*informed and confident in making choices that work for me and my family’* and more confident in their own decisions around breastfeeding. Some also reflected on how their increased confidence promoted positive help-seeking behaviours. One online media user referred to how:

[I would] seek out the help I need’ and how ‘[I would now] happily speak to someone if I am having any more issues. [Online Media/DIBM] (Participant 744).

Reassurance was evident in terms of how service users frequently commented that they felt ‘*relieved’, ‘calmer’*, and ‘*much better after the call’*. The NBH provided reassurance at critical points, for example, late at night when there were few other sources of support, when they had ‘*a query which felt really urgent’*, when having a ‘*difficult time trying to establish breastfeeding’* or when they were feeling vulnerable in what some described as ‘*the most vulnerable times of your life (4 days after birth)’.*

### Follow-up support

Volunteers suggesting that service users re-contact the NBH services as needed was a significant factor of overall satisfaction for the helpline callers (*p* = 0.002) only (Table 4). A few of the responders referred to how the support had continued to help them at various stages in their journey from the earliest of days to maintaining exclusive feeding and to maintaining breastfeeding into the postnatal period:

NBH Helpline has been a breastfeeding journey saver for me on several occasions where I have been struggling and have nearly given up altogether! I have not had any support regarding BF from the NHS etc. I simply internet searched for support and called, and received exceptional support and advice I will continue to use this service over and over in my BF journey and have and will continue to recommend to other breastfeeding mums. [Helpline] (Participant 216).

The NBH was described as *‘somewhere to turn to’* and how this was particularly beneficial when callers were ‘*at breaking point’*.

## Discussion

In this paper, we have considered the differences in service user and call/contact characteristics between those who access helpline or online media forms of support as well as what factors influence overall satisfaction for the different forms of support provided by the NBH service. Overall the findings showed differences in the demographics of those using the helpline or online media, and similarities and differences in what factors contribute to overall satisfaction when using different support modalities. Research on the efficacy and use of remote forms of breastfeeding support is growing [20] but there remains a lack of research on women’s experience of support and satisfaction regarding these forms of support [21]. This is important as while the need for remote breastfeeding support increased during the COVID-19 pandemic [7], the economic pressures on healthcare, and variability in breastfeeding support provision [22] indicate that this need will continue. Recent research has focused largely on comparing in-person support with that provided virtually [23] or on exploring one specific form of digital support, most commonly social media forums [15, 24–26] which may or may not be moderated by trained peer supporters or health care professionals. Our findings therefore address a key gap in terms of understanding who is using different forms of remote breastfeeding support, and what features appear to be most important.

When comparing the variables in terms of which services women chose to access, online media users were more likely to be younger, White, multiparous and less educated when compared to those who contacted the telephone helpline. These findings align with wider literature in terms of younger parents being more ‘technology savvy’, and are more accustomed to, and prefer interacting online [24, 27, 28]. Our findings support this by suggesting that online media can provide a useful source of support for parents in this cohort. Non-native English speakers were also more likely to use the helpline when compared to online media. These insights suggest that confidence to converse in English, rather than write in English, is perhaps more essential for helpline users when compared with online media. However, there are questions concerning the demographics of women who access NBH support. In our study, we found that 67.85% (78.03% with missing data removed) of service users were White British, with 79.48% (90.86% with missing data removed) having English as their first language. While the NBH offers helplines in four other languages the take up for these services is small and we were unable to access women who had used these services in this evaluation. While it is possible to argue that virtual support may offer improved access for some marginalised communities who have previously been less likely to take up in-person support, there remain challenges to using this approach to increase access for previously marginalised communities [25, 26]. Digital poverty can negatively affect communities’ ability to access this support [21] and further work is needed to ensure that equitable and acceptable support is provided [23, 29].

Overall, data indicated that there were similar factors that influenced satisfaction across both models of support. These factors were, the service being easy to access, receiving helpful information that met expectations and which helped to resolve their breastfeeding issues and for service users to feel reassured and more confident. These findings are congruent with a body of research with highlights that optimal breastfeeding support needs to be accessible, flexible, and instrumental [4, 30]. Furthermore, as service users’ views on service characteristics explained more variance in the regression modelling (for both forms of support), compared to the factors in other domains, this indicates that *how* support is provided is crucial for service users’ satisfaction. While there were issues about delays in calls being answered, and voicemails not being returned, this is likely indicative of the pressures of running a service run by volunteers, and with a limited budget [31]. Furthermore, while over a third of helpline callers felt that the opening hours should be extended, particularly when many new parents can experience nighttime breastfeeding challenges [4], this would require sufficient resources and infrastructure. The findings also reflect other research that suggests that face-to-face [4, 8, 10] and remote support can have a positive impact on breastfeeding experiences [25, 32]. The fact that online media was easier to access than the helpline is supported by other research which suggests that online support is more convenient as it can fit with personal and domestic situations [7, 21, 33].

In comparing the factors between the services, what also mattered for helpline users was support with cognitive and emotional-based issues of feeling that they were more knowledgeable about breastfeeding after the call/contact, feeling understood, more able to translate the information into practice, feeling encouraged to continue breastfeeding and seeking out additional support as needed. These findings suggest that as those who contact online media were more likely to request help with a specific concern, the focus may have been on self-management, whereas those using the helpline valued more in-depth, relational and translatable real-time support. This finding is also supported by the regression modelling which showed that variables in the helpline model explained more of the variance of satisfaction when compared to online media, across all three domains of factors. While there were issues concerning a lack of information about the different types of remote support available, these insights are likely to reflect that women access helplines when in ‘crisis’ mode and often when other support is unavailable [4, 10]; and that first-time mothers, who are more likely to experience breastfeeding challenges [34, 35], are more likely to contact the helpline rather than online media. Indeed some online support users noted that they would have called the helpline or sought help elsewhere if their situation had been ‘urgent’. When comparing these findings with the previous evaluation of the NBH, very similar factors were identified regarding ease of access, whether the information was helpful, resolved the breastfeeding issues, encouraged them to continue to breastfeed and met expectations and made them feel more confident and reassured [11].

The strengths of this study relate to a large data set that included qualitative feedback to help understand how the support was experienced. Individuals from a range of socioeconomic backgrounds participated, but further targeted work with younger, and non-White populations is needed. Dissemination of the NBH survey for those who contacted the helpline relied on volunteers collecting and forwarding information to the evaluation team (and for the call record to be updated) but in only ~ 50% of occasions was it clear whether service users’ details were collected, with 20% of occasions having no information whether the individual was notified or not. While details of the evaluation were meant to be forwarded to everyone who contacted the NBH social media/online options –response rates were very low for web chat and particularly the Drugs in Breastmilk service, which suggests this was not routinely undertaken. However, this could be due to service users wanting to remain anonymous, being very distressed during the call, or the call ending abruptly or not being particularly positive, which may have led to a potential bias in who participated. There were also challenges in accessing the views of those who used the non-English language helplines due to translation delays and then surveys not being completed, indicating that further ways to engage with callers using these services are needed. As participants were free to answer whatever questions they wanted, rather than forced choice (in line with ethical requirements to ensure the voluntary nature of participation), the number of missing responses towards the end of the survey increased, with between 10 and 11.1% of respondents not answering later questions.

## Conclusion

The nature of infant feeding support has changed over the last decade with the increased use and accessibility of remote breastfeeding support. Our study is the first to identify and compare factors that influence overall satisfaction amongst those who use different forms of remote support – helpline or online media. Our findings highlight that these different forms of support are needed to suit different demographics and call purposes, and that sufficient funding is needed to ensure a well-promoted and sustainable service can be provided. While optimal infant feeding support needs to be accessible, flexible and instrumental, helpline users are more likely to be first-time parents who need real-time relational support to deal with complex challenges. While remote support is suggested to provide a useful source of support for marginalised communities, there needs to be further work to ensure the nature, format and promotion of these resources to these communities. Further research that accommodates more acceptable ways to ensure that the views of minoritised populations are captured and included in service development is needed.

## Data Availability

All relevant data is included in the manuscript. A copy of the full evaluation report is available by contacting the lead author.

## Abbreviations

ABM: Association of Breastfeeding Mothers
BfN: The Breastfeeding Network
DIBM: Drugs in Breastmilk
NBH: National Breastfeeding Helpline
